# Identification of genes associated with cisplatin resistance in human oral squamous cell carcinoma cell line

**DOI:** 10.1186/1471-2407-6-224

**Published:** 2006-09-15

**Authors:** Ping Zhang, Zhiyuan Zhang, Xiaojian Zhou, Weiliu Qiu, Fangan Chen, Wantao Chen

**Affiliations:** 1 Department of Oral & Maxillofacial Surgery, Ninth People's Hospital, School of Medicine, Shanghai Jiao Tong University, Shanghai, 200011, China; 2 Shanghai Key Laboratory of Stomatology, Shanghai, 200011, China; 3 Department of Oral and Maxillofacial Surgery, School of Medicine, New York University, New York 10016, USA

## Abstract

**Background:**

Cisplatin is widely used for chemotherapy of head and neck squamous cell carcinoma. However, details of the molecular mechanism responsible for cisplatin resistance are still unclear. The aim of this study was to identify the expression of genes related to cisplatin resistance in oral squamous cell carcinoma cells.

**Methods:**

A cisplatin-resistant cell line, Tca/cisplatin, was established from a cisplatin-sensitive cell line, Tca8113, which was derived from moderately-differentiated tongue squamous cell carcinoma. Global gene expression in this resistant cell line and its sensitive parent cell line was analyzed using Affymetrix HG-U95Av2 microarrays. Candidate genes involved in DNA repair, the MAP pathway and cell cycle regulation were chosen to validate the microarray analysis results. Cell cycle distribution and apoptosis following cisplatin exposure were also investigated.

**Results:**

Cisplatin resistance in Tca/cisplatin cells was stable for two years in cisplatin-free culture medium. The IC50 for cisplatin in Tca/cisplatin was 6.5-fold higher than that in Tca8113. Microarray analysis identified 38 genes that were up-regulated and 25 that were down-regulated in this cell line. Some were novel candidates, while others are involved in well-characterized mechanisms that could be relevant to cisplatin resistance, such as *RECQL *for DNA repair and *MAP2K6 *in the *MAP *pathway; all the genes were further validated by Real-time PCR. The cell cycle-regulated genes *CCND1 *and *CCND3 *were involved in cisplatin resistance; 24-hour exposure to 10 μM cisplatin induced a marked S phase block in Tca/cisplatin cells but not in Tca8113 cells.

**Conclusion:**

The Tca8113 cell line and its stable drug-resistant variant Tca/cisplatin provided a useful model for identifying candidate genes responsible for the mechanism of cisplatin resistance in oral squamous cell carcinoma. Our data provide a useful basis for screening candidate targets for early diagnosis and further intervention in cisplatin resistance.

## Background

Head and neck squamous cell carcinoma (HNSCC) is a major public problem, frequently associated with devastating functional and cosmetic consequences for patients. More than 500,000 new cases are estimated to occur worldwide every year [1] and two thirds of patients present with locally advanced lesions and/or regional lymph node involvement. The benefits of chemotherapy for patients with advanced head and neck squamous cell carcinoma, demonstrated by recent meta-analyses of randomized studies, include reduction of the distant metastasis rate, improved survival rate and preservation of organ function, whether or not combined with local/regional treatment [2]. Cisplatin is one of the most potent chemotherapeutic agents currently in use, exerting its cytotoxic action through the formation of intra-strand DNA crosslink adducts [3]. However, the therapeutic benefits of apoptosis resulting from cisplatin-induced DNA damage can be attenuated, and the resistance that ensues is a major limitation of cisplatin-based chemotherapy. The molecular mechanisms underlying the acquisition of resistance to cisplatin are not fully understood. Multiple mechanisms have been described in gastric, colonic and ovarian cancer cells [4-6]. It is believed that the molecular signature defining the cisplatin-resistant phenotype differs among tumors and generally involves many factors. In order to elucidate the cisplatin resistance mechanisms in oral squamous cell carcinoma, we established a cisplatin-resistant cell model with progressively acquired chemoresistance, Tca/cisplatin, which was derived from a cisplatin-sensitive cell line. We used Affymetrix HG-U95Av2 microarrays to analyze the differences in gene expression patterns between this resistant cell line and its sensitive parent line, with the aim of identifying genes associated with cisplatin resistance in this subtype of HNSCC. When maintained in cisplatin-free culture medium for two years, Tca/cisplatin still preserved a stable cisplatin-resistant character. Compared with previously reported cell lines, analysis of these Tca/cisplatin cells disclosed some novel drug-resistance associated genes [12-14].

## Methods

### Establishment of cisplatin-resistant cells

Cisplatin resistance in Tca/cisplatin, a variant cell line derived from Tca8113, was developed by exposure to cisplatin for 24 months, starting at 1 μM and ending at 10 μM. Despite massive cell death among the sensitive Tca8113 cells under treatment, the cultures were maintained by regular changes of medium and intermittently increasing the cisplatin concentration until the surviving cells recovered a normal growth pattern. Before testing, the Tca/cisplatin cells were continuously maintained in cisplatin free RPMI-1640 medium (Invitrogen, CA) supplemented with 10% fetal bovine serum (Gibco, USA) for two years.

### Growth inhibition

Growth inhibition was determined by a MTT assay repeated six times. In brief, cells were seeded in 96-well plates at a density of 2 × 10^3 ^cells/well (200 μl/well) for 24 h before use. The culture medium was replaced with fresh medium containing different concentrations of cisplatin ranging from 0 to 160 μM for 48 h. Water-soluble tetrazolium MTT (Sigma-Aldrich, USA) was added (20 μl). After a further 4 h incubation, the supernatant was discarded and the purple crystals were re-suspended in 200 μl of DMSO. The absorbance of each well was read at 570 nm on an ELISA XL (BIOHIT, BP800, Finland). Growth rate was calculated as the ratio of the absorbance of the experimental well to that of a blank well. The IC50 (concentration of drug that results in 50% of control value) was calculated as previously described [7].

### Apoptosis assay

Apoptosis induced by treatment with different concentrations of cisplatin for 48 h was assayed using an Annexin V-FITC/PI Apoptosis Detection Kit (BD Pharmingen, USA) following the manufacturer's protocol; assays were repeated three times. The results were analyzed using Cell Quest (BD Pharmingen, USA) and flow cytometry (FACScalibur, Becton-Dickinson Co., USA) was used to distinguish the cells as viable (Annexin V-/PI-), early apoptotic (Annexin V+/PI-) or late apoptotic (Annexin V+/PI+).

### Cisplatin accumulation

For cisplatin accumulation experiments, cells were exposed to 200 μM cisplatin for 2 h and then harvested. The drug exposure was relatively brief, so that the contribution of net platination of DNA could be isolated and the effect of DNA repair reduced, as discussed previously [8]. Total DNA was extracted. Platinum concentrations were determined using Inductively Coupled Plasma Atomic Emission Spectrometry (ICP, Thermo Jarrell Ash, IRIS Advantage 1000, USA) at the Department of Instrumental Analysis Center of Shanghai Jiao Tong University; measurements were repeated three times.

### RNA preparation and gene expression

Total RNA was extracted and analyzed on HG-U95Av2 Affymetrix oligonucleotide arrays (Affymetrix, CA, USA) containing 12,626 probe sets for human genes, as described previously [9]; analyses were repeated three times. Gene expression profiles were compared between Tca/cisplatin and Tca8113, with Tca8113 cells as baseline, using GeneChip Suite 5.0 software (Affymetrix, CA, USA). All the genes represented on the microarray were globally normalized and scaled to a signal intensity of 500. Fold changes were calculated by comparing transcripts between the sensitive and the acquired-drug-resistant cell lines. In GeneChip Suite 5.0 software, Wilcoxon's test was used to identify detected (present or absent) and changed (increased or decreased) calls. This provided a basis for statistical determination of whether or not a transcript was expressed and whether it was relatively increased, decreased or unchanged.

### Real-time PCR

Several interesting genes including *MAP2K6, RECQL, CCND1, CCND3, ABCB1*, *ABCC2 *and *GST-Pi *were selected to validate the results of microarray analysis, as described previously [10]. Total RNA was extracted and reverse transcribed to cDNA (Life Technologies, USA). The primer sets and product lengths are listed in table 1. PCR was carried out according to the standard protocol of SYBR Premix Ex Taq (TaKaRa, Japan) using Real-time PCR Equipment (ABI 7300, ABI, USA). To quantify changes in gene expression, the ΔΔCt method was used to calculate the relative fold changes, normalized against glyceraldehyde-3-phosphate dehydrogenase.

### Western blotting

Whole-cell extracts were prepared in cell lysis buffer (M-Per, Pierce, USA). The first antibodies were: mouse monoclonal anti-CCND1 (Cell Signaling, clone DCS6, dilution 1:2000), rabbit polyclonal anti-CCND3 (Proteintech Group, dilution 1:1000), mouse monoclonal anti-ABCB1 (Calbiochem, clone C219, dilution 1:1000) and mouse monoclonal anti-GST-Pi (Abcam, clone BD1340, dilution 1:1000). Signals were visualized using Supersignal West Pico Chemiluminescent Substrate (Pierce). Mouse monoclonal anti-β-actin (Sigma, clone AC-15, dilution 1:10000) was used throughout as loading control.

### Immunohistochemical staining

Fresh specimens of primary oral squamous cell carcinoma were collected aseptically from the Department of Oral & Maxillofacial Surgery, Ninth People's Hospital, between June 2003 and July 2005. The specimens were minced with scissors and passed through a 200 pores/cm^2 ^steel mesh to prepare tumor cell suspensions. Each suspension was cultured with or without 10 μM CDDP for 72 h. Growth inhibition was evaluated using a modified MTT assay to assign each sample to the cisplatin-resistant or the cisplatin-sensitive group [11,12]. Immunohistochemical staining was performed on formalin-fixed, paraffin-embedded, 4 μm-thick tissue sections using the standard SP method. First antibodies were: mouse monoclonal anti-CCND1 (Cell Signaling, clone DCS6, dilution 1:100) and rabbit polyclonal anti-CCND3 (Proteintech Group, dilution 1:100). The staining intensity in each section was evaluated by three pathologists and graded according to the average percentage of positive cells in three randomly selected fields: – (0–5% cells stained), + (5%-20% cells stained), ++ (20%-50% cells stained) and +++ (>50% cells stained).

### Cell cycle analysis

Cells in exponential growth were cultured in 10 μM cisplatin for 6, 12 or 24 h, then washed twice in PBS and fixed in 70% cold ethanol overnight. Cell pellets were resuspended in 1 mg/ml RNase (Sigma-Aldrich, USA) at 37°C for 30 min, followed by staining with 50 μg/ml propidium iodide (Sigma-Aldrich, USA). For each sample, 10^4 ^cells were analyzed by flow cytometry (FASCalibur, BD, USA). Results were expressed as the percentage of cells in each phase of the cell cycle (Mean ± SD).

### Statistics

An ANOVA test was used to examine the statistical differences in growth inhibition and apoptosis rates between Tca/cisplatin and Tca8113 cells. Student's *t *test was used to determine the statistical differences between these two cell lines in cisplatin accumulation and Real-time PCR results. Immunohistochemical staining results were analyzed by a Mann-Whitney U test. *P *< 0.05 was considered statistically significant. A significance analysis of microarrays algorithm (SAM) was used to identify significant differences in gene expression between these two cell lines (triplicate results), with the estimated false discovery rate (FDR) setting as 1 and the response type setting as two class unpaired.

## Results

### Cisplatin-resistant phenotype of Tca/cisplatin cell line

The Tca/cisplatin cell line was obtained by stepwise selection from its sensitive parent cell line over a period of two years. At the beginning of induction, cell growth was strongly suppressed. However, at the end of induction, the derived cells proliferated fairly rapidly. After further maintenance for up to 60 passages in cisplatin-free RPMI1640 supplemented with 10% fetal bovine serum, the resistant cells exhibited stable biological characteristics: the population doubling time was 29.93 h, while that of the parent cell line was 38.82 h. The drug-resistant cells were stable for two years in cisplatin-free culture medium.

### Sensitivity to cisplatin

**Figure 1:**
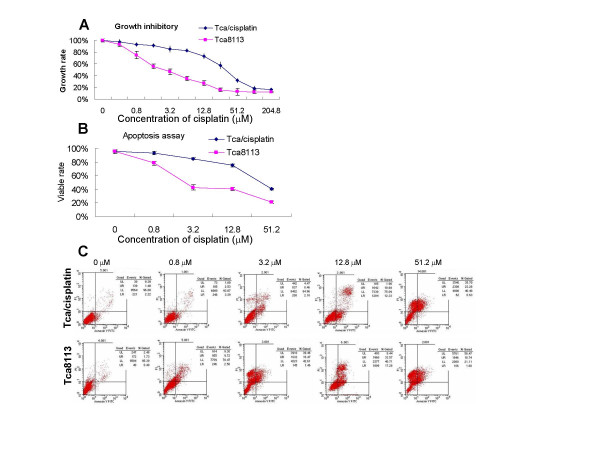
**Response of Tca/cisplatin and Tca8113 cells to cisplatin**. A: Dose-growth rate curve plotted from the MTT assay results. Both cell lines displayed dose-dependent growth inhibition, but the Tca/cisplatin cells were more resistant to the antiproliferative activity of cisplatin (*P *= 0.0000, *ANOVA *analysis). Each point was the mean of six independent experiments. B: Dose-viability curve was plotted from the Annexin-V/PI assay results. There was a dose-dependent decrease in viability in both cell lines, but the decrease was more rapid in the Tca8113 cells (*P *= 0.0000, *ANOVA *analysis). Each point was the mean of three independent experiments. C: Scatter plots of Annexin V/PI staining. As the cisplatin dose increased, the apoptosis rates (UR+LR) also gradually increased, but the increase was more rapid in Tca8113 cells than in Tca/cisplatin cells (*P *= 0.0000, *ANOVA *analysis). Each figure represents three independent experiments.

Cells were treated with different concentrations of cisplatin for 48 h and dose-response curves were plotted as shown in Figures 1A and 1B. Dose-dependent antiproliferative activity and apoptosis (Figure 1C) were observed in both cell lines; however, the resistance of Tca/cisplatin to cisplatin was 6.5 fold higher than that of the parental Tca8113 cells, as measured by the IC50 values for cisplatin over 72 h treatment [13]: 26.69 ± 3.42 μM with 95% confidence interval [19.46, 38.43], and 4.11 ± 1.21 μM with 95% confidence interval [2.07, 7.14], respectively.

### Cisplatin accumulation in Tca/cisplatin and Tca8113 cells

**Figure 2:**
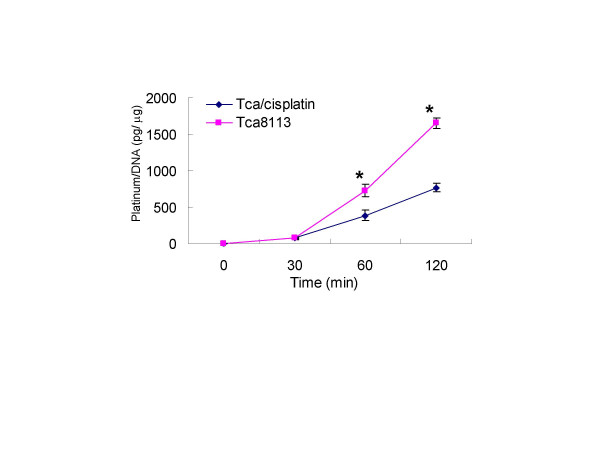
**Platinum accumulation on DNA in Tca/cisplatin and Tca8113**. DNA was extracted from Tca/cisplatin cells (blue) and Tca8113 cells (pink) after treatment with 200 μM cisplatin for 0, 30, 60 and 120 min. The platinum concentration in isolated DNA was measured by ICP. Each point is the mean of three independent experiments. Significant differences were observed at 60 min (*P *= 0.008, Student's *t* test) and 120 min (*P *= 0.002, Student's *t *test), which are marked with asterisks.

Intracellular accumulation of cisplatin was compared between Tca/cisplatin and Tca8113 cells by determining the amount of platinum in DNA. Time-dependent accumulation was observed (Figure 2), and after 2 h exposure, Tca8113 had 2.15 times as much DNA-associated cisplatin as Tca/cisplatin cells.

### Different gene expression profiles in Tca/cisplatin and Tca8113

The Affymatrix HG-U95Av2 microarray contains 12626 probe sets, most of which have previously been characterized in term of function or disease association. In order to distinguish valid signals from background noise, three replicates were performed using independently isolated RNA samples for each cell line, and a total of 6 hybridizations were analyzed. Scatter plots of the normalized signals were constructed and the average of the correlation coefficients for replicates was 0.951, suggesting good consistency. SAM was used to identify genes that were significantly differently expressed between these two cell lines, with FDR setting = 1. As shown in Table 2, a total of 63 probe sets were selected, among which 38 genes were up-regulated and 25 down-regulated in the Tca/cisplatin cell line. On the basis of the Gene Ontology list, these 63 genes were primarily classified according to potential function into metabolism-related genes, cell cycle-related genes, transcription factors, substrate transporters, signal transduction-related genes, oncogenes, and so on. The down-regulated genes included those involved in cell cycle arrest, regulation of cell proliferation, nucleic acid binding and protein metabolism, while the up-regulated genes mostly included those involved in cell cycle regulation (*CCND1, ASNS*), DNA repair (*RECQL*), small molecule non-selective transport (*ITPR1*), protein synthesis (*GARs*), transcription (*ID1, ID2*) and oncogenes (*c-syn, FGFR3, RAB31*). The up-regulated genes could result in cell cycle acceleration, increased proliferation, enhanced DNA metabolism and synthesis, altered molecular transport and elevated transcriptional activity; Tca/cisplatin had a shorter population doubling time (29.93 h) than the parental cell line (38.82 h).

### Confirmation of differential expression by Real-time PCR and Western blotting

**Figure 3:**
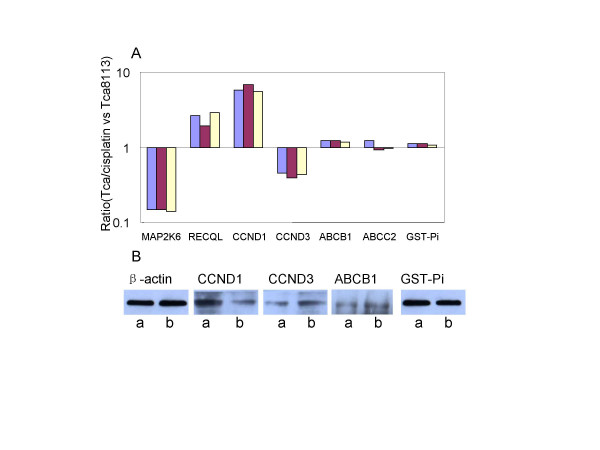
**Validation of genechip results by Real-time PCR and Western blotting**. A: Using Real-time PCR, we validated the expression of *MAP2K6, RECQL, CCND1, CCND3, ABCB1, ABCC2 *and *GST-Pi *transcripts in both cell lines and calculated the relative fold changes by normalizing against *GAPDH *expression. Ratios of transcripts in Tca/cisplatin cells and Tca8113 cells generally showed the same expression differences as microarray analysis. Each column represents the results of an independent experiment (repeated three times). B: Using Western blotting, we further validated the expression of CCND1, CCND3, ABCB1 and GST-Pi proteins in both cell lines, using β-actin as a loading control. The results also show alterations similar to those found using the genechip. Each figure represents three independent experiments. (a: Tca/cisplatin cells, b:Tca8113 cells).

Seven genes – *MAP2K6 *in the MAP pathway, the DNA repair-related gene *RECQL*, the cell cycle-related genes *CCND1 *and *CCND3*, ATP-binding cassette family members *ABCB1 *and *ABCC2*, and the cellular detoxification-related gene *GST-Pi *– were selected for further verification of the microarray results by Real-time PCR. Also, the expression of CCND1, CCND3, ABCB1 and GST-Pi proteins was further tested by Western blotting, exploiting their functional linkages to biochemical mechanisms that could be related to cisplatin resistance. As shown in Figure 3, which represents the results of three independent experiments, both the Real-time PCR (Figure 3A) and Western blotting (Figure 3B) analyses confirmed the microarray results.

### Cell cycle distribution

**Figure 4:**
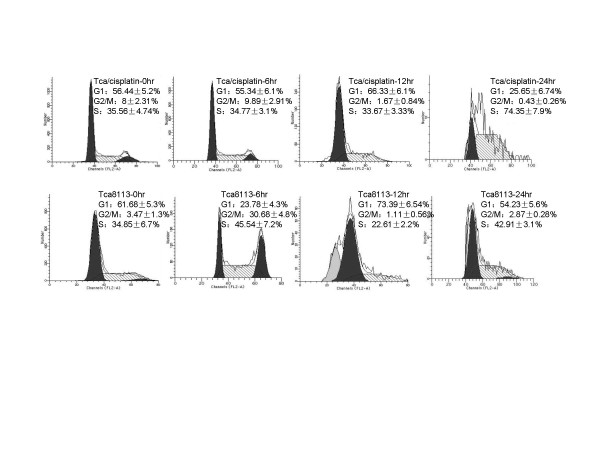
**Effects of 10 μM cisplatin on Tca/cisplatin and Tca8113 cell cycle distribution**. After exposure to 10 μM cisplatin for 0, 6, 12 and 24 h, cells were harvested and stained with propidium iodide to assess cell cycle distribution by FACS analysis. Representative time course for Tca/cisplatin and Tca8113 cells in different phases of the cell cycle (one time course each) are shown. The results are expressed as mean ± SD of three independent experiments. A predominant S phase block of 74.35% was observed at 24 h in the Tca/cisplatin cells, significantly higher than the 42.91% in the Tca8113 cells.

Analysis of the gene expression profiles suggested that the cell cycle-related genes *CCND1 *and *CCND3 *were differently expressed in Tca/cisplatin and Tca8113 cells. We therefore analyzed the cell cycle distribution further following cisplatin exposure. As shown in Figure 4, prolonged exposure to cisplatin for 6, 12 or 24 h induced a significant difference in cell cycle distributions in the two cell lines. A predominant S phase block was observed at 24 h in the Tca/cisplatin cells; this was 74.35%, significantly higher than the 42.91% in the Tca8113 cells.

### Expression of CCND1 and CCND3 in primary oral squamous cell carcinoma

**Figure 5:**
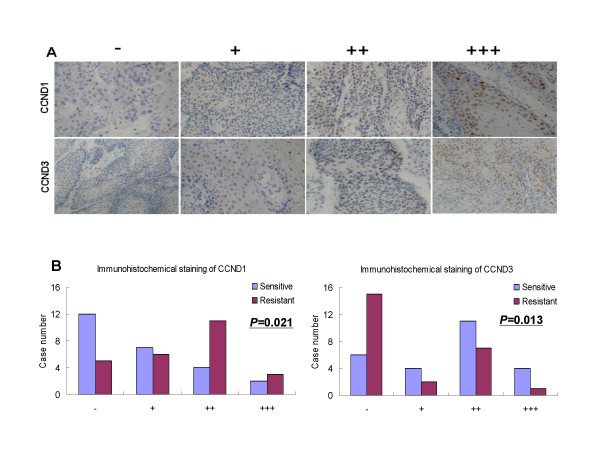
**Expression of CCND1 and CCND3 in primary oral squamous cell carcinoma**. Fifty specimens of primary oral squamous cell carcinoma were collected and classified into resistant (red column) and sensitive (blue column) groups on the basis of growth inhibition as assessed by the modified MTT assay, 25 cases in each group. A. Immunstaining results were ranked according to the staining percentage: '-' (0–5%), '+'(5%-20%), '++' (20%-50%) and '+++' (>50%). B. Mann-Whitney U test showed that CCND1 (*P *= 0.021) and CCND3 (*P *= 0.013) were differentially expressed in primary tumors in a manner consistent with their drug resistance patterns.

Clinical samples were classified into cisplatin-sensitive and resistant groups on the basis of growth inhibition as indicated by the MTT assay [11]. The cutoff point for the sensitive group was above 50%, while for the resistant group it was below 30% [12]. Samples with growth inhibition rates between 30%-50% were excluded to eliminate bias. In total, 50 samples qualified for inclusion in the final analysis, 25 in each group. As Figure 5 shows, CCND1 and CCND3 staining was mainly observed in the nuclei. Using the scoring method described above for CCND1 staining, 48% of samples in the sensitive group were graded as '-', 28% as '+', 16% as '++' and 8% as '+++'; while only 20% in the resistant group were graded as '-', 24% as '+', 44% as '++' and 12% as '+++'. For CCND3 staining, 24% of samples in the sensitive group were graded as '-', 16% as '+', 44% as '++' and 16% as '+++'; while in the cisplatin resistant group 60% were '-', 8% '+', 28% '++' and 4% '+++'. In other words, up-regulation of CCND1 and down-regulation of CCND3 were observed in the resistant group whereas the opposite changes were noted in the sensitive group. The Mann-Whitney U test confirmed statistically significant differences in CCND1 (*P *= 0.021) and CCND3 (*P *= 0.013) between these two groups.

## Discussion

Here we established an isogenic cisplatin-resistant variant from the oral squamous cell carcinoma cell line Tca8113 so that we could compare their gene expression profiles directly. It is clear from our results that the cisplatin-resistant cell line, Tca/cisplatin, has a genome-wide expression profile that differs from the Tca8113 line. Our microarray analysis results revealed 63 differently expressed genes, including some previously reported genes that have been related to cisplatin resistance (*CCND1, ID1, ID3, GCA*, etc.), and also some novel ones such as *TRIM29, N33, GRAS *and *GPI *[14-16]. Considering that Tca/cisplatin and Tca8113 cells have identical genetic backgrounds, we supposed that these differently expressed genes are related to cisplatin resistance and the differences might shed light on the mechanism of resistance to this chemotherapeutic agent. Nevertheless, extensive investigation of the detailed effects of these genes in conferring the cisplatin-resistance phenotype on oral squamous cell carcinomas is needed to provide potential strategies for reversing the resistance [16,17].

It is well known that cisplatin cytotoxicity is attributable to the formation of various DNA adducts that trigger a cellular response culminating in apoptosis. Correspondingly, the major resistance mechanisms that limit the extent of DNA damage include reduced drug uptake by the ABC transporter, increased drug inactivation by cellular detoxification, increased DNA adduct repair by DNA repair systems, etc. [18]. We observed a 2.15-fold higher cisplatin accumulation in Tca8113 cell DNA than in Tca/cisplatin cell DNA after 2 h treatment with cisplatin. We further investigated the expression of *ABCB1*, which is the first ABC transport identified [19], *ABCC2*, which mediates the active efflux of glutathione-conjugated cisplatin [20], and *GST-Pi*. No difference was found between our cell lines by SAM analysis of the microarray results, which were further validated by Real-time PCR. However, over-expression of *RECQL*, a DNA helicase involved in various types of DNA repair including mismatch, nucleotide excision and direct repair, was increased in our cisplatin-resistant cells, though this is the first suggestion that it is associated with cisplatin resistance [21]. An enhanced DNA repair rate would also attenuate the apoptotic process induced by the formation of DNA adducts of cisplatin, which has been demonstrated in several studies in murine and human tumor cell lines [22-26]. Because our samples were cultured under cisplatin-free conditions for 2 years, the lack of difference in *ABCB1, ABCC2 *and *GST-Pi *expression may also reflect their characteristics as transient drug-resistance genes.

Cell cycle arrest is an initial cellular response to DNA damage. After treatment with 10 μM cisplatin for 24 h, a marked S phase block was observed in Tca/cisplatin cells but not in Tca8113 cells. Cell cycle arrest is necessary to enable the nucleotide excision repair complex to remove the DNA adducts and to promote cell survival. Only when repair is incomplete, as would be the case when damage is extensive, will cells undergo apoptosis [27]. Cisplatin-induced S phase block may be involved in DNA adduct repair in cisplatin resistant cells, which has also been reported in the cisplatin-resistant A2780-cis cell line when compared with sensitive A2780 cells [28]. Owing to the complex relationship between cell cycle arrest and cytotoxicity, the underlying mechanisms of cell cycle arrest and drug resistance have not been fully deciphered. Cell cycle progression is strictly regulated by the activation of a series of cyclins and cyclin-dependent kinases [29]. Among these, CCND1 is a cell cycle regulatory factor that modulates a critical step in cell cycle control. In Tca/cisplatin cells, an interestingly altered pattern of expression of the cell cycle genes *CCND1 *and *CCND3 *was found: CCND1 was up-regulated while CCND3 was down-regulated. CCND1 and CCND3 usually show overlapping expression patterns, yet their functions are not totally redundant; for example, in human mammary epithelial cells, CCND1 expression peaks in G1 and declines before the S phase, while CCND3 expression rises later in G1 and remains elevated in S phase [30]. CCND1 is preferentially associated with cyclin-dependent kinases 4 and 6, which inactivate the retinoblastoma tumor suppressor protein via phosphorylation, thereby overcoming the Rb-mediated blockade of G1 and facilitating G1-S progression. Overexpression of CCND1 has been linked to tumor resistance to cisplatin in fibrosarcoma cell lines and in breast, pancreatic, colon and lung cancers [31]. Wang and colleagues [32] also reported that antisense *CCND1 *enhances the sensitivity of head and neck squamous cell carcinoma cells to cisplatin. So it was reasonable to hypothesize that the inverse expressions of CCND1 and CCND3 might participate in the cisplatin resistance of Tca/cisplatin cells. Furthermore, our preliminary results from 50 oral squamous cell carcinoma samples showed that the inverse expression pattern of CCND1 and CCND3 correlates with cisplatin sensitivity.

The measurement of gene expression can provide information on regulatory mechanisms, biochemical pathways, cellular control mechanisms and potential targets for intervention and therapy in a variety of disease states [15]. Our results support the hypothesis that multiple specific genes contribute to the development of cisplatin resistance in squamous cell carcinoma. Microarray analysis of the model system for differentially expressed genes involved in cisplatin resistance in squamous cell carcinoma has revealed a variety of genes, including some putatively common drug-resistance-related genes, and this provides a rational basis for determining which pathways are appropriate for further study and which molecular targets are potential targets for gene therapy [16,17]. Further exploration based on these findings will provide new insights into the complicated molecular events of drug resistance. Having determined the existence of such differences, the next question is: to what extent are these gene expression alterations related to cisplatin-resistance? There may be heterogeneity in the gene expression profiles among malignant tumors of a particular histopathological grade and chemotherapeutic agent resistance potential. Studies of the molecular mechanisms regulating these changes would help to identify prognostic biomarkers and treatment targets for HNSCC chemotherapy.

## Conclusion

The Tca/cisplatin and Tca8113 cell lines are useful models for identifying candidate genes responsible for the mechanism of cisplatin-resistance in oral squamous cell carcinoma. Sixty-three genes related to cisplatin-resistance were identified. Among these, decreases in cell cycle arrest genes and increase in oncogenes, cell cycle regulation gene and genes involved in metabolism and synthesis led to the cell cycle acceleration, increased proliferation rate and resistance to cisplatin-induced apoptosis in Tca/cisplatin cells. CCND1 and CCND3 seemed to be closely involved in cisplatin resistance. The data from this study provide useful clues to screen candidate targets for early diagnosis and intervention in cisplatin-resistance.

## Competing interests

The author(s) declare that they have no competing interests.

## Authors' contributions

PZ carried out the molecular genetic and cellular phenotype studies, performed the statistical analysis and drafted the manuscript. ZYZ conceived of the study and participated in its design and coordination. ZXJ participated in establishing cell lines and carried out the immunoassays. WLQ participated in the study design. FAC carried out the microarray testing and performed the statistical analysis. WTC conceived of the study, participated in its design and coordination and helped to draft the manuscript. All authors read and approved the final manuscript.

## Pre-publication history

The pre-publication history for this paper can be accessed here:



## Figures and Tables

**Table 1:** Primer lists of Real-time PCR

Gene Name	Primer Sequence	Product
MAP2K6	5' GAGTCTGGGCATCACG 3'	103bp
	5' CGATGGCTCCTCTACC 3'	
RECQL	5' CAATGGCTGGAAAGGAGGTA 3'	112bp
	5' AATGGGCAAATGACGAGTGT 3'	
CCND1	5' GCTGGAGCCCGTGAAAAAGAG 3'	326bp
	5' GGATTGGAAATGAACTTCACA 3'	
CCND3	5' CTGCCTCCAGGAACCACA 3'	146bp
	5' GCTTGACTAGCCACCGAAAT 3'	
ABCC1	5' AAATGCAGCTGATGAATCCAAAA 3'	271bp
	5' TTGCAAATGCTGGTTGCA 3'	
ABCC2	5' CACTTCAGCGAGACCG 3'	246bp
	5' GGACAGAACAAAGCCAAC 3'	
GST-pi	5' TTTCGCCGCCGCAGTCT 3'	122bp
	5' TCCACGGTCACCACCTCCTC 3'	
GAPDH	5' AACGGATTTGGTCGTATTGGG 3'	516bp
	5' AGTCTTCTGGGTGGCAGTGAT 3'	

**Table 2:** Genes differently expressed in cisplatin resistant SCC cells

Classification	Gene	Change Fold ^a^
Metabolism	AKR1C3	-3.96
	ALDH3B1	-2.14
	GPI	-2.00
	NNMT	-2.00
	MAOB	-2.00
	NAGA	2.14
	FLJ12443	2.14
	SLC27A2	2.30
	SLC2A3	2.83
	GARS	2.00
	QPRT	2.14
Cell cycle	CCND3	-2.83
	CCND1	4.29
	ASNS	2.83
Transcript factor	TRIM29	-2.00
	ZFP36	-2.83
	CREM	2.83
	ETV5	4.29
	ID1	2.00
	ID3	4.00
	CAMTA2	2.00
Transport	MUC1	-2.14
	ATP1B1	-2.00
	TCIRG1	-2.30
	COX8	-2.46
	KPNB2	2.30
Signal transduction	MAP2K6	-2.30
	IGFBP3	-5.66
	PDLIM1	2.00
	IGFBP7	2.30
	PDE8B	2.14
	IL13RA1	3.03
	ARF6	2.14
	GCA	7.06
	PIP5K1A	2.00
	ITPR1	2.64
Oncogene	FYN	2.83
	FGFR3	3.03
	RAB31	2.83
	EMS1	-2.14
Others	CHST2	-2.83
	C1S	-2.83
	H2AFO	-2.30
	PPL	-2.30
	TGM2	-2.30
	KIAA0992	-2.14
	SELENBP1	-2.00
	H2BFB	-2.00
	DKFZp564J0323	-2.00
	PON2	-2.00
	FUSIP1	2.00
	DDOST	2.00
	ODAG	2.14
	RECQL	2.14
	DMD	2.14
	G1P2	2.30
	RAFTLIN	2.30
	SEMA3F	2.30
	N33	2.30
	TUBA3	2.30
	KRT7	2.46
	BC008967	3.25
	ITGA1	3.48

a: mean of triplicate measurements.
